# Single pixel hyperspectral bioluminescence tomography based on compressive sensing

**DOI:** 10.1364/BOE.10.005549

**Published:** 2019-10-07

**Authors:** Alexander Bentley, Jonathan E. Rowe, Hamid Dehghani

**Affiliations:** 1 School of Computer Science, College of Engineering and Physical Sciences, University of Birmingham, UK; 2 Physical Sciences for Health Doctoral Training Centre, College of Engineering and Physical Sciences, University of Birmingham, UK

## Abstract

Photonics based imaging is a widely utilised technique for the study of biological functions within pre-clinical studies. Specifically, bioluminescence imaging is a sensitive non-invasive and non-contact optical imaging technique that is able to detect distributed (biologically informative) visible and near-infrared activated light sources within tissue, providing information about tissue function. Compressive sensing (CS) is a method of signal processing that works on the basis that a signal or image can be compressed without important information being lost. This work describes the development of a CS based hyperspectral Bioluminescence imaging system that is used to collect compressed fluence data from the external surface of an animal model, due to an internal source, providing lower acquisition times, higher spectral content and potentially better tomographic source localisation. The work demonstrates that hyperspectral surface fluence images of both block and mouse shaped phantom due to internal light sources could be obtained at 30% of the time and measurements it would take to collect the data using conventional raster scanning methods. Using hyperspectral data, tomographic reconstruction of internal light sources can be carried out using any desired number of wavelengths and spectral bandwidth. Reconstructed images of internal light sources using four wavelengths as obtained through CS are presented showing a localisation error of ∼3 mm. Additionally, tomographic images of dual-colored sources demonstrating multi-wavelength light sources being recovered are presented further highlighting the benefits of the hyperspectral system for utilising multi-colored biomarker applications.

## 1. Introduction

Bioluminescent Imaging (BLI) is a widely used modality within pre-clinical biomedical studies. It is a highly sensitive and non-invasive technique that can detect distributed biological visible and near-infrared light sources from for example, luciferase-catalyzed reaction [1], which allows for a non-invasive method of detecting and visualizing functional activity within live intact animals. BLI has been shown to have the ability to track cells around the body, including potential sanctuary sites such as the brain [2]. The light signal from the luciferase-catalyzed reaction increases during the first minutes, reaching a plateau after 10 to 15 minutes, after which the signal remains fairly constant for ∼40 minutes [3]. The characteristics of the bioluminescent signal therefore give a safe timeframe for imaging within 20–30 minutes after the luciferin injection [4].

Although highly specific, current limitations to this method include poor spatial resolution and the quantitative accuracy of the information that bioluminescent imaging provides, as it is known to be affected by the difficulties in implementing it efficiently. As bioluminescent signals have very low intensities, and are non-linearly attenuated by the often unknown underlying tissue optical attenuation, highly-sensitive spatially resolved detectors are required. To allow the analysis from BLI to be more quantitative, methods that allow for the recovery of spatially resolved tomographic maps of the bioluminescent source location and intensity can be employed, known as Bioluminescent Tomography (BLT) [5]. In BLT, a ‘forward’ model of light propagation from the internal source to the surface of the subject, along with an optimization recovery ‘inversion’ algorithm are used to reconstruct the underlying source spatial and intensity distribution. There are several issues that arise with current BLT systems, including the non-uniqueness of single wavelength data [6]. To overcome this, multi-wavelength data of the emission at the surface of the subject due to the bioluminescence is collected by using spectrally resolved detection schemes such as bandpass filters, however this causes the time used to collect data to increase as data from individual filters have to be collected sequentially. Another issue is due to the effects of filter bandwidth on the quantitative accuracy of BLT [7], which has been shown to have dramatic effects on the reconstruction quality and is also often difficult to control due to the limited bandwidth of filters available. A final challenge to overcome is that most existing approaches only take into account the propagation of light from the light source to the surface of the subject and not from the surface of the subject to the optical detector. To address this, it is possible to either model the light propagation in free-space using ray-tracing techniques [8] or to utilise spectral derivative data [9].

Compressive Sensing (CS) is a method of signal processing that utilizes the sparse nature of real world signals in order for them to be compressed either in its original domain or in some transformed domain. It works in a similar way to standard image/signal compression algorithms such as JPEG-2000, where a data vector which represents the raw pixels of the image is transformed using the discrete wavelet transform (DWT). Once the image has been transformed, all of the small wavelet coefficients are set to zero leaving behind a sequence that can be stored efficiently and when required later can be inverse-transformed to provide an approximate representation of the original image or signal [10]. This technique finds the basis or domain of a signal that is sparse or compressible, meaning that a signal of length *n* can be represented by k≪n nonzero coefficients. A sparse signal can then be represented with high accuracy by only keeping the values and locations of the largest coefficients of the signal.

By using this concept of CS, it is possible to create a new framework for both acquiring signals and how sensors are designed. If a signal is sparse or compressible, it is possible to acquire a signal with less samples than is classically suggested within the Nyquist-Shannon sampling theorem, which states there needs to be a minimum number of measurements taken in order to perfectly capture an arbitrary signal. Using this approach, rather than first sampling at a high rate and then compressing the collected data, it is possible to directly collect the compressed data. This enables a potentially dramatic reduction in the sampling and computational costs of measuring signals that are sparse [11], as is the case in BLI and BLT.

CS has been applied to a number of applications within the area of biomedical imaging, such as Diffuse Optical Tomography (DOT) and Fluorescence Molecular Tomography (FMT). Recent work has demonstrated the application of CS into a multiple view DOT/FMT system, which is based on structured light illumination, compressive detection and multiple view acquisition. Two digital micro mirror devices (DMD) are utilized for illumination and acquisition is carried out using a time resolved single pixel detector. The system was validated using a tissue-mimicking phantom and demonstrated good agreement with data obtained using a CCD method [12]. Single-pixel imaging has been be used in a wide variety of applications as shown by Edgar et al [13], however is has not yet to date been demonstrated in BLT [14–16].

By applying the basis of CS to the application of BLT, it is possible to incorporate cheaper single dimensional (in space) detectors to allow for the collection of hyperspectral data. By using a single-pixel acquisition allows for the collection of hyperspectral data which in turn will potentially improve tomographic recovery, sensitivity and specificity particularly for multi-colored sources which is the main motivation behind this work [17]. Collecting data this way would potentially bring improvements to the issues outlined, such as non-uniqueness and the bandwidth size, as these are both highly tunable when using a spectrally resolved detector. This is a novel approach of hyperspectral imaging and has the potential to be faster and cheaper than existing hyperspectral cameras as these often use a line scanning method and can cost upwards of $50000. Existing schemes typically collect data using non-contact systems utilizing a CCD camera with filters which are pre-defined for wavelengths and to improve data acquisition typically have large bandwidths. Collecting hyperspectral data using existing schemes is unfeasible due to the length of acquisition time required, further motivating the proposed methodology. In this work, the development of a compressive sensing based hyperspectral Bioluminescence tomographic imaging system is presented. Preliminary results using this system are shown utilizing block and mouse phantoms containing single internal artificial light sources and multiple light sources of different wavelengths.

## 2. Theory

**Fig. 1.**
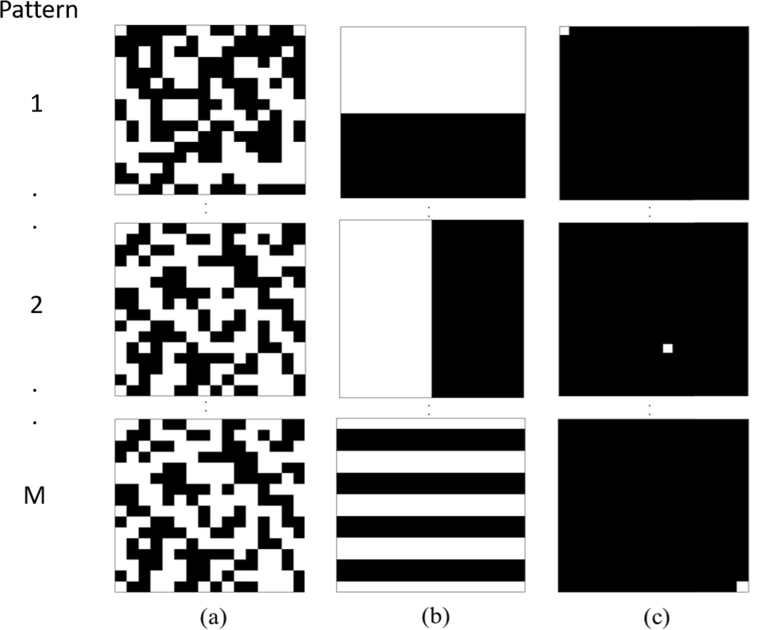
Examples of the different types of measurements matrices that can be used for image compression. (a) Randomly generated patterns with a Gaussian distribution, (b) Hadamard matrices and (c) a raster scanning method where the scene is scanned pixel by pixel.

By considering an N × 1 signal *x*, that is real-valued, finite-length and one-dimensional, it is possible to represent this as the basis of N × 1 vectors $\{\varphi_{i}{\}}_{i=1}^{N}$. Using this, the signal *x* can be expressed as,

(1) $$x=\sum_{i=1}^{N}s_{i}\varphi_{i}=\varphi s$$

where *s* is an N × 1 column vector of weight coefficients. The signal *x* is defined as K-sparse if only K of the *s_i_* coefficients are nonzero, and if K<<N the signal is compressible. In order to directly capture the compressed signal, M < N linear measurements are taken of the inner products of *x* and a collection of vectors $\{\phi_{j}{\}}_{j=1}^{M}$ such that $y_{j}=\langle x,\phi_{j}\rangle$. Arranging *y_j_* into a M × 1 vector *y*, $\phi_{j}$ as rows in an M × N matrix ϕ and substituting *x* from Eq. (1), *y* can be written as,

(2) y=ϕx=ϕφs=Θs

where Θ is an M × N matrix. If the measurements *y* are collected for a certain measurement matrix ϕ, there are two conditions that need to be overcome in order to accurately obtain the signal *x*. Firstly, the measurement matrix needs to be designed so that important information within the signal is not lost by the dimensionality reduction from N to M measurements. Secondly, a reconstruction algorithm needs to be designed so that it can correctly recover the signal *x* from only M measurements [18]. When collecting data, the measurement matrix is designed to be constructed of 1’s and −1’s that is randomly generated. For this to work in practice the data is collected using a measurement matrix formed from 1’s and 0’s and is subsequently corrected using a pattern that is full. Additionally for the problem to be well conditioned it needs to have restricted isometry property (RIP), which states that the length of the matrix Θ must be the same length as the as the K-sparse vector being measured. Another condition that has to be met is incoherence, meaning that the rows of ϕ cannot sparsely represent the columns of φ. Figure 1 shows different measurement matrices that can be used to collect the compressed data used for image reconstruction. The first column shows Gaussian distributed random matrices and the second shows a binary pattern taken by sampling rows of the Hadamard matrix. Both of these matrices have been shown to have both incoherence and meet the restricted isometry property, so can be used in this application [19]. The third column of Fig. 1, represents the patterns used when raster scanning the subject, where a measurement is taken for each pixel of the reconstructed image, hence taking full measurements and not utilizing CS. Further types of measurement basis can also be used such as wavelet, noiselet and speckle patterns depending on which basis the signal is sparse [13].

The conventional method for signal reconstruction from the measured ‘compressed’ data, is to find a solution to Eq. (2) by formulating it as a minimization problem:

(3) $$\min\left(\|y-\Theta s{\|}_{2}^{2}+\lambda^{2}\|s{\|}_{2}^{2}\right)$$

where λ is the regularization parameter and $\left\|.\right\|{\;}_{2}$ is the L_2_ norm. The solution to Eq. (3) has a simple closed form, however will not induce sparsity into the solution and will often return a non-sparse solution with many nonzero values. In order to maximize the sparsity induced into the solution, minimizing the L_p_ norm for p ≤ 1 can be used. Using the L_0_ norm will induce sparsity more strongly as it essentially counts the number of nonzero values of the solution, however it is both numerically unstable and NP-complete, so is extremely difficult to minimize [20]. Instead, minimizing the L_1_ norm of vector *s* can both find the correct K-sparse solution and be done using computational algorithms such as primal dual methods [21], Nesterov’s method [22] and conjugant gradient methods [23]. This then becomes the construction of a linear convex optimization problem:

(4) $$min\|s{\|}_{1}\;\;\;such\;that\;\;\Theta s=y$$

If it is assumed that instead of the signal being sparse, the gradient of the underlying signal or image is sparse, as is the case in both BLI and BLT, it is possible to recover the signal by minimizing the total variation (TV) of the signal instead of the conventionally used L_1_-norm,

(5) $$\min\underset{i}{\sum }\|w{\|}_{i},\;such\;that\;\phi x=y;\;D_{i}x=w_{i}$$

where $D_{i}x$ is the discrete gradient of *x* at pixel *i*. Using TV-regularization over L_1_-regularisation can result in the reconstructed images being sharper due to the edges and boundaries being preserved more accurately. TV regularization has been used extensively since its introduction in 1992 by Rudin, Osher and Fatemi for its use in image denoising [24]. Since then it has been used in many other applications such as image deconvolution [25] and image restoration [26]. One issue with TV regularization is that the properties of non-differentiability and non-linearity make it much more computationally difficult than L_1_-norm regularization. One method to solve this problem is to rewrite the constrained problem as a sequence of unconstrained sub problems, as is the case in the total variation minimization by augmented lagrangian and alternating direction algorithm (TVAL3) that has been utilized in this work [27,28] and detailed extensively elsewhere [29].

## 3. Methods and results

### 3.1. Hyperspectral imaging system

**Fig. 2.**
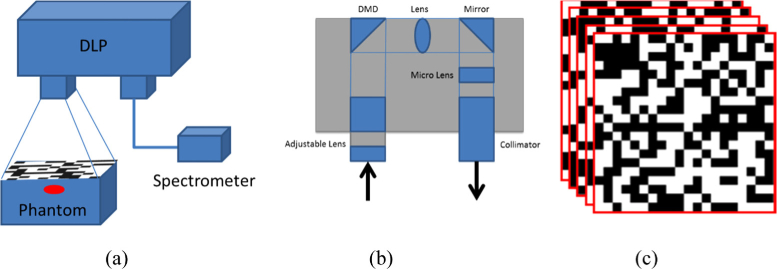
(a) Schematic of the developed hyperspectral imaging system, (b) Schematic of the internal components within the DLP used and (c) the random binary patterns that are displayed on the digital micro-mirror device.

Figure 2 presents a schematic of the imaging system that has been developed, showing the different components that allow measurements from a sparse source distribution map. A Texas Instruments DLP Lightcrafter 4500 has been modified so that the digital micro-mirror device (DMD) within can be used to direct random projections of the imaging scene into a spectrometer. The DLP is modified by removing the three LEDs that are part of the system and then attaching one end of an optical fiber in its place for detection. The optical fiber used in this work has a core diameter of 1000 µm. The DMD within the DLP has an array of 912 by 1140 micro mirrors that can be individually controlled to be in either an ‘on’ or ‘off’ position. This allows for random binary patterns to be created as shown in Fig. 2(a). The spectrometer used in the system is a Flame S-VIS-NIR (OceanOptics), which has an optical detection range of 350 nm to 1000 nm with a spectral resolution of 0.4 nm, which is suitable as the wavelengths detected for a typical BLI are typically around 600 nm. It contains a 200 µm slit and uses a Sony ILX511B linear silicon CCD array to detect the incident light. Both the DLP and the spectrometer are controlled using MATLAB that automatically collects data once the desired resolution, number of measurements and acquisition time have been selected. The system includes an adjustable stage that the object being imaged can be placed on to correct and set the imaging field of view and focus. The whole system fits within a custom made light-proof housing to eliminate any background light, increasing the signal-to-noise ratio.

### 3.2. Effect of the number of measurements on image reconstruction accuracy

An experiment was undertaken to demonstrate the impact of the number of random patterns M, used in data collection on the accuracy and quality of the image reconstructions. Spectral data was collected as described above. The imaged object was a block phantom (Biomimic, INO, Quebec, Canada) of dimensions 33 × 26 × 40 mm. The phantom is made of a solid plastic with homogeneous spectrally-varying optical absorption and scattering properties that have been characterized within the range of 500 to 850 nm in terms of the absorption coefficient, µ_a_ = [0.007–0.12] mm^−1^, and the reduced scattering coefficient, µ_s_´ = [1.63–1.79] mm^−1^ [30]. The phantom body contains two tunnels with a diameter of 6 mm at depths of 5 mm and 15 mm in which rods of matching optical properties to the background can be inserted to create a solid homogeneous phantom. A rod containing a light source was made that can be inserted into either of the two channels to mimic a bioluminescent light source. The light source used to mimic in-vivo bioluminescence in the experiment was a standard 5 mm LED (Arduino), with the emission spectrum being a Gaussian-like curve with a central peak at 620nm and a full-width-half-maximum of ∼20 nm, meaning it has a similar spectral output to a bioluminescent reporter.

**Fig. 3.**
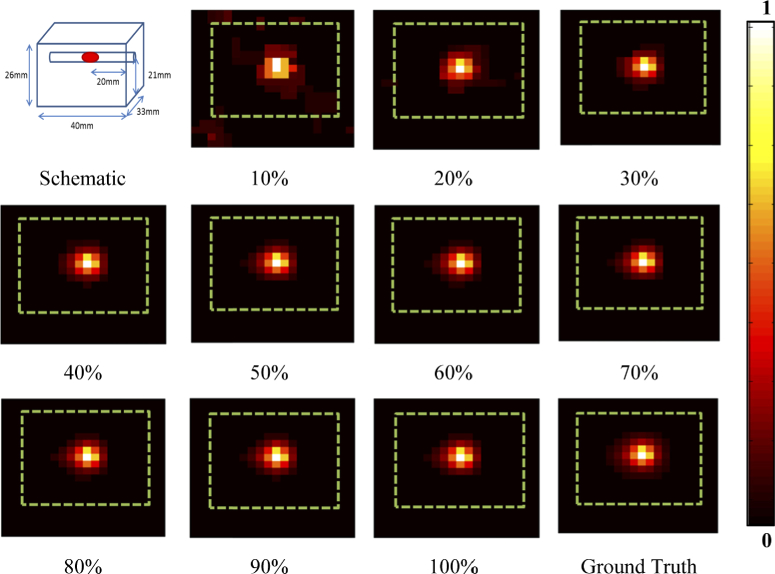
Schematic of the phantom setup and the reconstructed surface fluence images for the number of measurements (as a percentage of total pixels) used for reconstruction at 620 nm. Green dashed line represents the outline of the phantom. The ‘ground truth’ image was measured using a raster-scan of 400 measurements.

For the experiment the light source was placed at a depth of 5 mm inside the block phantom. This was then imaged by sequentially collecting the spectral data of the imaging scene convolved with a series of binary patterns that are displayed on the DMD within the imaging system at an acquisition time of 200 ms per pattern. The binary patterns used in this experiment are a series of 400 randomly generated 20×20 pixel patterns made up of ones and zeros. After the spectral data had been collected a total variation minimizing algorithm [31] was used to reconstruct images of the surface light fluence of the phantom using 10% to 100% of the total amount of measurements M, at a wavelength of 620 nm with a bandwidth of 5 nm. The percentage of measurements used for reconstruction is a percentage of the total amount of pixels of the reconstructed image, for example when reconstructing a 20×20 image reconstructing using 10% measurements will be using 40 measurements. The reconstructed images were exported in Tagged Image Format (TIF) and the recovered photon intensity as a function of number of measurements used is shown in Fig. 3. It is clear from the images that as the number of measurements used for reconstruction is increased, they more closely represent the ground truth, which is captured using a raster scanning method (i.e. 400 individual measurements for each pixel).

**Fig. 4.**
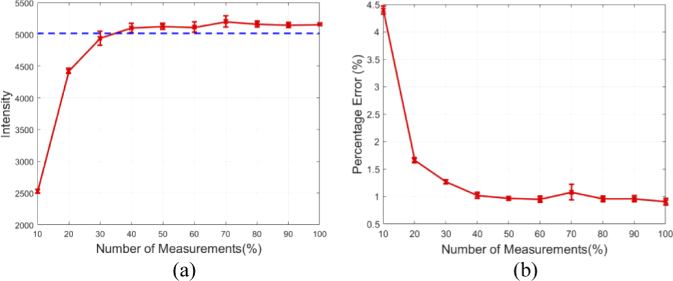
(a) Maximum reconstructed value for the number of measurements used for reconstruction (solid red) and the maximum value for the ground truth (dashed blue). (b) The percentage error between the reconstructed images and the ground truth for the number of measurements taken.

To better represent the accuracy and quality of the surface fluence reconstructions, the detected maximum intensity of the signal for each image presented in Fig. 3 is plotted in Fig. 4(a) with respect to the percentage number of measurements used, as compared to the full raster scan ‘Ground Truth’ image. Data collection was repeated four times in all experiments and the standard deviation of the data is shown in the error bars. It can be seen that when using a low number of measurements (<30%, M < 120) the maximum intensity is lower than the ground truth, whereas at higher number of measurements (>30%) the maximum intensity asymptotes at a value similar to the ground truth. The percentage error of the reconstructed images as compared to the ground truth has also been plotted as a function of the number of measurements used for reconstruction in Fig. 4(b). It can be seen that at a similar point as the asymptote in Fig. 4(a), there is an asymptote at a percentage error of ∼1%. These findings have shown that it is possible to reconstruct an image of the fluence data at the surface of the subject, using as low as 30% of the total number of measurements taken when using a standard raster scanning method whilst maintaining quantitative accuracy. However, for the remainder of this work, a total of 50% (i.e. M = 200) of measurements will be used.

### 3.3. Effect of the measurement matrix ‘fullness’ on image reconstruction accuracy

**Fig. 5.**
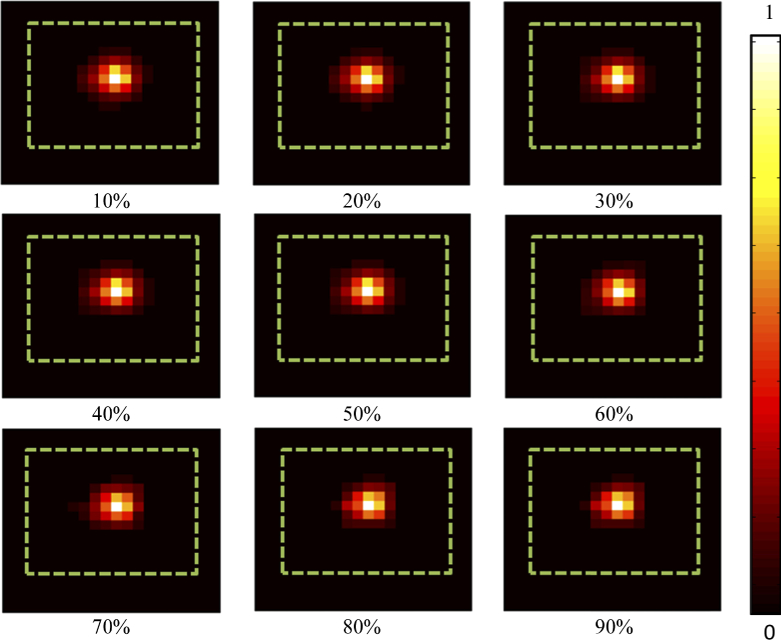
The surface fluence images reconstructed at a wavelength of 620 nm for different measurement matrix ‘fullness’. Green dashed line represents the outline of the phantom.

A second experiment was undertaken to demonstrate the effect of the ‘fullness’ of the measurement matrix used to collect the spectral data. Although the effect of fullness of the pattern has been previously investigated in depth, this effect has not been investigated for random patterns [32]. The term ‘fullness’ describes the percentage of 1’s used in the binary patterns that are displayed on the DMD within the system, Fig. 1(a). The term ‘fullness’ used in this work is only applicable to the random binary patterns used as this is not switchable for other well defined basis. Increasing the ‘fullness’ of the measurement matrix will improve the signal-to-noise ratio that is obtained as it increases the amount of spatial information being sampled, however it may affect the quality of the reconstructions of the surface fluence. For this experiment the setup is the same as in the previous section where a tissue mimicking block phantom is used with an LED of peak wavelength of 620 nm as the light source. Spectral data of the imaging scene as obtained with the binary patterns was collected as before. The light source was placed at a depth of 5mm and the spectral data was collected using M = 200 (i.e. 50% of total measurements) 20×20 binary patterns at an acquisition time of 200 ms per pattern, which were then repeated for varying measurement matrix ‘fullness’. The same total variation minimizing algorithm was then used to reconstruct images of the surface fluence with varying ‘fullness’ at 620 nm with a bandwidth of 5 nm. The reconstructed images were exported in TIF and the detected photon intensity as a function of number of measurements used is shown in Fig. 5.

From the images it can be seen that there is little qualitative differences in the reconstructions between different matrix ‘fullness’, with slight variations apparent in the higher ‘fullness’ percentages (70%–90%).

**Fig. 6.**
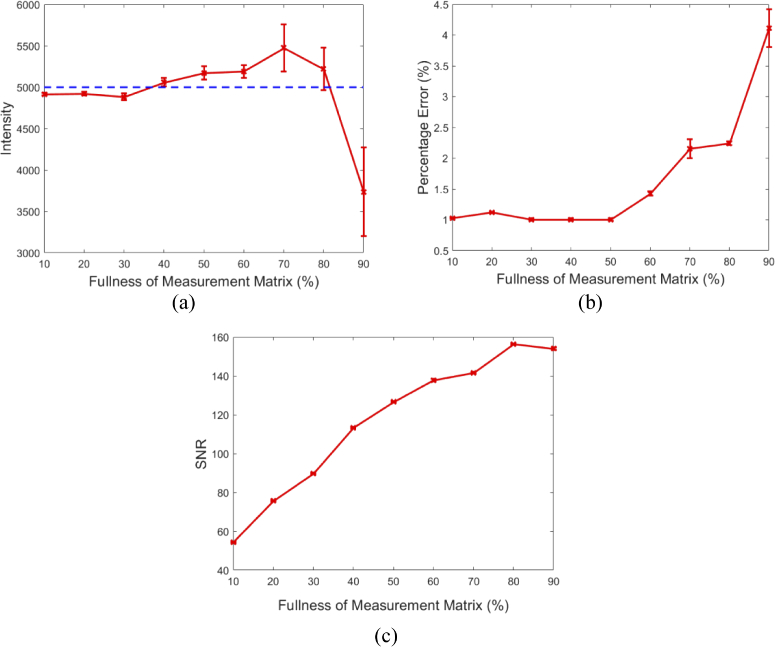
(a) Measured maximum reconstructed value for the measurement matrix ‘fullness’ used for reconstruction (solid red) and the maximum value for the ground truth (dashed blue). (b) The percentage error between the reconstructed images and the ground truth for the measurement matrix ‘fullness’ used for reconstruction. (c) The signal-to-noise ratio obtained for each measurement matrix ‘fullness’ used.

To quantitatively analyze the images, the maximum reconstructed intensity is plotted in Fig. 6(a) with respect to the matrix ‘fullness’. It can be seen that the maximum intensity of the reconstructions closely resembles that of the ground truth at 10% up to 60%, after which, the variation in the reconstructions become large. It is believed that this relationship is due to sparseness of the measurement matrix and not meeting the restricted isometry property (RIP) that is required for a compressive sensing based method to work. To further quantitatively analyze the images, the percentage error as compared to the ground truth (as from Fig. 3.) is plotted in Fig. 6(b) with respect to the matrix ‘fullness’. The same pattern as with the maximum intensity can be seen, where the error is constant at 1% for the lower percentage ‘fullness’, whereas at a matrix ‘fullness’ of >60% the percentage error of the reconstructions increase and the quality decreases. Finally, the peak signal-to-noise ratio (SNR) of the measured data is plotted in Fig. 6(c) with respect to the measurement matrix ‘fullness’. It can be seen that the SNR of the measured data increases linearly as the matrix ‘fullness’ increases as would be expected. However, due to the effects of higher percentage ‘fullness’ on the maximum intensity and percentage error, the increase in SNR does not provide a benefit in image reconstruction. These findings however are case specific due to the reconstruction quality being dependent on the size and sparsity of the signal, therefore greater or less sparse signals will show different reconstruction qualities.

### 3.4. Tomographic reconstruction using a tissue mimicking block phantom

**Fig. 7.**
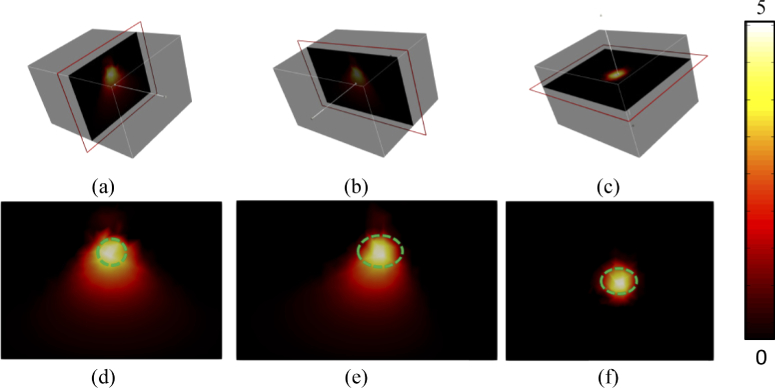
2D cross-sections of the tomographic reconstruction of a 620 nm LED within the block phantom from the (a, d) front view, (b, e) side view and (c, f) top view.

Using the information gained from the analysis of the data collected in the previous two sections, an experiment was performed to tomographically reconstruct the spatial light distribution of a light source within a tissue mimicking block phantom. Using the same optical setup as defined previously, spectral data was collected sequentially using M = 200 (50% of total pixels) binary patterns with ‘fullness’ of 50% and an acquisition time of 200 ms. Surface fluence images of the phantom at four different wavelengths (610 nm, 620 nm, 630 nm, and 640 nm) were reconstructed using the same total variation algorithm as used previously. The wavelengths were selected with a bandwidth of 10 nm and covered the majority of the emission spectrum of the LED being used. The images were then registered to a model of the phantom and normalized. The fluence images were then utilized together with NIRFAST which is an open-source Finite Element model-based image reconstruction package for diffuse optics and molecular imaging (www.nirfast.org). Within the NIRFAST, a compressive sensing based optimization algorithm has been developed that uses a forward model of light propagation through the phantom based on the diffusion approximation of the radiative transport equation [23,33]. The solution found is the spatial distribution of the light source and can be visualized as 2D cross-sections of the 3D model, Fig. 7.

Qualitatively there is good accuracy in the localization of the source in the reconstruction as compared to the ground truth. To quantify the accuracy of the reconstructions, firstly the volume of the source was calculated at full width half maximum and secondly the location of the center of mass of the source was calculated, Table 1.

**Table 1.** The expected and measured volume of the internal light sources and the localization error of the reconstructed sources.

	Actual Volume (mm^3^)	Measured Volume (mm^3^)	Localization Error (mm)
Block Phantom	137.0	72.0	4.0
Mouse Phantom	50.2	54.7	3.7
Block (RED)	137.0	88.5	3.0
Block (GREEN)	137.0	123.0	2.7

### 3.5. Tomographic reconstruction using a tissue mimicking mouse phantom

**Fig. 8.**
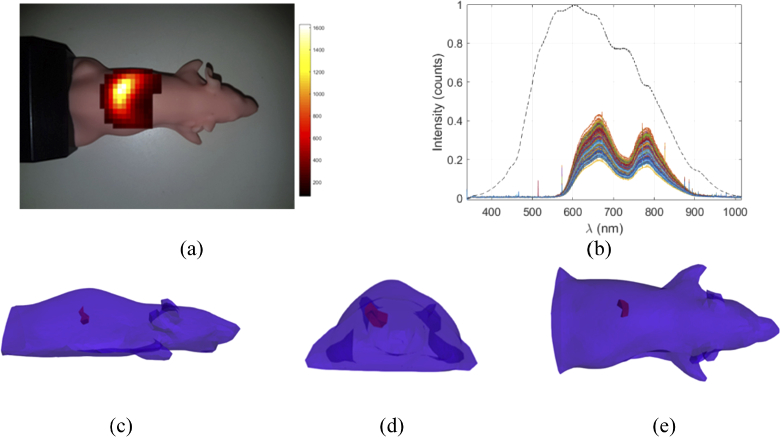
(a) Overlaid image of the recovered surface fluence of the fiber light source inside the mouse shaped phantom. (b) The hyperspectral data collected from the mouse phantom, each colored line represents a measurement using one pattern, the black dashed line represents the emission spectrum of the internal light source. Tomographic reconstruction of the light source from the (c) side, (d) front and (e) top.

To demonstrate the application of this technique on a more geometrically realistic model, a mouse shaped phantom (XFM-2, Perkin Elmer Inc., Waltham, MA, USA), Fig. 8(a), embedded with an optical fiber connected to an Ocean Optics HL-2000 halogen light source to mimic a light source with dimensions of 4 mm x 4 mm x 4 mm and a peak emission of 620 nm, Fig. 8(b). The phantom is made from polyurethane material that includes scattering particles and dye to simulate the optical properties of live tissue, which have been characterized to have an absorption coefficient, µ_a_ ≈ 0.01 mm^−1^ and a reduced scattering coefficient, µ_s_´ ≈ 1.5 mm^−1^ at a wavelength of 600 nm. The light source was placed in a channel within the phantom at a depth of 10mm. Hyperspectral emission data due to the light source at the surface of the phantom was collected using the same method as described previously, with M = 200, 20×20 pixel binary patterns with a ‘fullness’ of 50% and an acquisition time of 200ms. Surface fluence images were reconstructed from the data at four wavelengths (610 nm, 620 nm. 630 nm and 640 nm) with a bandwidth of 10 nm, which were then normalized and registered to a model of the phantom, for tomographic reconstruction, Fig. 8(a).

The 3D spatial light distribution was reconstructed from the recovered surface fluence using NIRFAST utilizing the same compressive sensing based optimization algorithm as in the previous section. The raw reconstructed surface fluence image at 620 nm and the tomographic reconstruction of the light source are shown in Fig. 8. It was found that the spatial distribution of the light source was reconstructed with good localization (<3 mm) and volume accuracy, Table 1.

### 3.6. Tomographic reconstruction of multiple sources of different wavelengths

A final experiment was undertaken to demonstrate an additional and otherwise difficult benefit of collecting hyperspectral data, for the reconstruction of the spatial light distribution of multiple light sources with different peak emission wavelengths using the same data set. The imaged object in this experiment was the block phantom used previously. Two different LEDs (Arduino) were used as a light source, one with a peak emission 620 nm (Red) and one with a peak emission of 510 nm (Green). Data was collected using the same method as before, by measuring the spectral data of the surface emission due to the light source using a sequence of M = 200 20×20 binary patterns of 50% ‘fullness’ and an acquisition time of 200ms. Surface fluence images were reconstructed from the spectral data at four different wavelengths for each source, which were 500 nm, 510 nm, 520 nm and 530 nm for the green source and 610 nm, 620 nm, 630 nm and 640 nm for the red source, all with a bandwidth of 10nm. The reconstructed fluence images were normalized and registered to a model of the phantom and the internal spatial light distribution was then reconstructed as before.

**Fig. 9.**
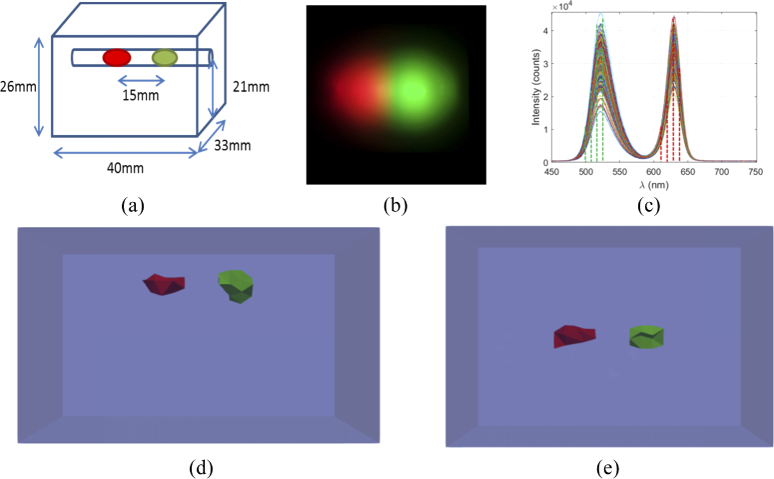
(a) Schematic of the phantom setup. (b) RGB color image of the reconstructed surface emission due to the internal LEDs. (c) Emission spectra of the red and green LEDs, the dashed lines represent the wavelengths that measurements were taken for tomographic reconstruction. *Each curve represents a measurement taken with an individual random binary pattern.* Tomographic reconstructions of the green and red LEDs from the (d) side and (e) top.

A RBG color image of the emission of the light sources at the surface of the phantom and the raw spectral data collected are shown in Fig. 9 as well as the reconstructed spatial light distribution of both sources. It can be seen that both light sources are reconstructed with good localization and volume accuracy, Table 1.

## 4. Discussions

The number of spectral measurements used to reconstruct an image of the emission at the surface of object due to an internal light source using a compressive sensing method, as described in this paper can greatly affect both the qualitative and quantitative accuracy of the reconstructions, Fig. 2. As described in literature, the minimum number of measurements required to accurately represent an image depends on both the number of pixels in the image and the underlying sparseness of the image [17]. In the experiments displayed within this work, using a typical example of an internal light source, it was found that the surface fluence of the light source could be accurately reconstructed to within 1% error of the ground truth using as low as 30% of the total number of pixels reconstructed in measurements. To further demonstrate the effect that the number of spectral measurements has on the accuracy of image reconstruction, the maximum intensity of the reconstructed value was evaluated against the number of measurements. It was found that the recovered maximum value was comparable to that of the ground truth when only 30% of the total measurements were used for reconstruction. This reduction in number of measurements directly relates to an imaging time that is at least 30% (due to increase SNR as compared to single pixel raster scanning) of the time taken in order to collect the same data using existing methods for hyper-spectral imaging, such as raster scanning. In pre-clinical studies it is common practice to associate the total count in intensity to be proportional to the total amount of activity occurring, therefore it is important that there is no variation in the detected data. Due to these finding it was concluded that 50% of the total reconstructed pixels in measurements would be used for all proceeding reconstructions.

The ‘fullness’ of the measurement matrix used in data collection was shown to have an underlying effect on the quality of the surface fluence images reconstructed from the spectral data that was collected, Fig. 3. It was found that using a matrix ‘fullness’ of ≤50% resulted in reconstructions that were within 1% error of the ground truth whilst also showing comparable maximum reconstructed values. When the measurement matrix ‘fullness’ was >50% the quality of the reconstructions were seen to reduce and the maximum reconstructed value shown to fluctuate from the ground truth with greater variation in measurements. It is shown in literature that in order for a compressive sensing based method to successfully find the solutions to the underdetermined problem that is present, the measurement matrix has to meet the restricted isometry property (RIP), which states that if the measurement basis of the matrix is too closely aligned to the sparsity basis of the measured signal it will be unable to detect the signal [34]. This property is typically met by using a randomly generated measurement matrix, however as the ‘fullness’ of the measurement matrix increases the RIP is no longer holding due to the randomness of the matrices being reduced. It was also found that as the ‘fullness’ was increased, the measured peak SNR linearly increased. The benefit of a higher SNR however is not valid for this application as the variation and quality of the reconstructed images is of greater importance. Therefore, it was concluded that a measurement matrix ‘fullness’ of 50% would be used for all proceeding experiments.

Tomographic reconstructions were made using both a block phantom and a mouse phantom using the hyperspectral compressive sensing based system that has been developed, Fig. 4 and Fig. 5. It was found in both cases that the spatial distribution of the light source was reconstructed with both good localization and volumetric accuracy that is comparable to values quoted in literature [35]. Tomographic reconstructions of multiple sources of different wavelengths have also been shown to be possible from the same set of collected spectral data. In this experiment both sources were reconstructed with good localization and volumetric accuracy, however reconstruction of the green source appeared to be more successful. This is believed to be due to the optical properties of the phantom that are used within the reconstruction algorithm being more closely matched to the true values for the green wavelengths as compared to the red wavelengths. This highlights the importance of obtaining a-prior information regarding the optical properties of the medium for in-vivo applications. It has previously been demonstrated that in a practical setting a multi-modal system [1] or ATLAS based information [9,36] can be used to estimate the optical properties of the tissue being imaged.

Using this CS based method to collect hyperspectral emission data from the surface of the imaging subject has the potential to address a number of issues that have previously been raised in respect to BLI and BLT. Firstly, by collecting hyperspectral data, all in one collection strategy, can help address the issue of the variation in measured signal as a function of imaging time [9]. This method can also combat the issue of non-uniqueness in the solution as it has been shown that collecting multi-spectral data improves the accuracy of tomographic reconstruction [6]. It has been demonstrated in previous studies that using multiple views is beneficial when imaging deep in vivo sources [1,9]. The method of data collection via compressive sensing as used in this work, is also applicable to multi view data collection and can be incorporated into system designs. There is the potential for the time taken for data collection of multi/hyperspectral data to be vastly reduced with this method. Another issue that can be addressed by this method is the effect of filter bandwidth on measurement as no filters are used [7]. The effective bandwidth of measurements is limited by the spectral resolution of the spectrometer, so can be controlled better [7], and is a topic for future work. Moving forward, it has been shown that using the spectral derivative of the spectral data measured will eliminate the need for any system corrections or system models as light at similar wavelengths display near-identical system responses [9,37]. This is also a future direction of development for the outlined imaging system, as this data can easily obtained from the hyperspectral measurements.

## 5. Conclusions

This work highlights the development of a hyperspectral compressive sensing based imaging system used for non-contact BLI and BLT. The effect of varying the number of measurements and ‘fullness’ of the measurement matrix has been explored and it has been shown that images made with 30% of the measurements taken in existing systems can be reconstructed with as little as a 1% error, as compared to the ground truth. The ground truth used in this work is collected using a pixel-by-pixel raster scanning scheme which is thought to provides definite information about each pixel in the image [13]. The system has also been shown to be able to carry out tomographic reconstructions using a mouse shaped phantom and a block phantom with both individual and multiple sources of two different wavelengths. Although the use of LED’s for these experiments are providing signals much stronger than those from a bioluminescent source, this work has demonstrated the application of CS in BLT paving the way for further optimization of the system to deal with lower light levels as seen in pre-clinical studies. This could be achieved through the development of a unique detection system, rather than an adapted off-the-shelf projector, a more sensitive spectrometer and overall optimization of the system to minimize signal loss due to coupling of the DMD and spectrometer. The system can be optimized by first improving the optical fiber that collects the light from the DMD by increasing the diameter of the fiber, decreasing the length of the fiber and decreasing its proximity to the DMD. A DMD that is optimized for better reflection with visible/NIR should also be used. Lenses with better transmission and shorter focal lengths will reduce the imaging distance, as for example a factor of 4 reduction in imaging distance will result in a factor of 16 increase in signal. Finally the spectrometer can also be configured to use a lower spectral resolution such as 2nm instead of 0.3nm to improve detection sensitivity*.* The use of adaptive patterns can also be explored to further speed up data collection whereby knowledge of the domain being imaged can be utilized [32]. These will provide a hyper-spectral system at a resolution not yet achieved, at potentially a lower cost, which will be applicable to multi-marker imaging in pre-clinical studies. As the proposed CS based system utilizes a ‘single’ pixel detection, the effective area for sensing light can be seen to increase as compared to utilization of a multi-pixel camera, providing better SNR at lower light levels which will be subject of further investigation.
